# Skeletal Muscle Effects of GLP 1 Receptor Agonists: Molecular Mechanisms and Comparative Insights on Semaglutide and Tirzepatide, a Narrative Review

**DOI:** 10.3390/ijms27188057

**Published:** 2026-09-10

**Authors:** Domenico Cautela, Valeria Incarbona, Angela Lombardi, Bruna Laratta, Curzio Massimo Castaldo, Maria Luisa Balestrieri, Edoardo Mocini, Silvia Migliaccio, Daniela Tardito

**Affiliations:** 1 Dipartimento di Scienze Teoriche e Applicate (DiSTA), Università Telematica eCampus, Via Isimbardi 10, 22060 Novedrate, CO, Italy; domenico.cautela@uniecampus.it (D.C.); valeria.incarbona@studenti.uniecampus.it (V.I.); angela.lombardi@uniecampus.it (A.L.); edoardo.mocini@uniecampus.it (E.M.); 2 Istituto di Bioscienze e Biorisorse (IBBR), CNR, Via Pietro Castellino 111, 80131 Napoli, NA, Italy; bruna.laratta@cnr.it; 3 Scuola di Medicina e Chirurgia, Università degli studi della Campania Luigi Vanvitelli, Via Santa Maria di Costantinopoli 16, 80138 Napoli, NA, Italy; curziomassimo.castaldo@studenti.unicampania.it; 4 Dipartimento di Medicina di Precisione, Università degli studi della Campania Luigi Vanvitelli, Via de Crecchio 7, 80138 Napoli, NA, Italy; marialuisa.balestrieri@unicampania.it; 5 Dipartimento di Medicina Sperimentale, Sapienza Università di Roma, Piazzale Aldo Moro 5, 00185 Rome, Italy; silvia.migliaccio@uniroma1.it

**Keywords:** GLP-1 receptor agonists, tirzepatide, semaglutide, skeletal muscle, sarcopenia, lean mass, dual GLP-1/GIP agonists, metabolomics

## Abstract

GLP-1 receptor agonists (GLP-1RAs), dual GLP-1/GIP agonists, and the more recent triple GLP-1/GIP/glucagon agonists have transformed the treatment of type 2 diabetes mellitus (T2DM) and obesity, resulting in significant weight reduction. However, a substantial proportion (20–40%) of this weight loss is attributable to a decrease in lean mass, thereby raising concerns regarding potential sarcopenia-related risk. However, decreased lean mass is not sufficient to determine sarcopenia, which is now defined by the combined presence of decreased muscle mass, diminished strength, and impaired physical function, particularly in older or frail individuals. Thus, a further clarification of the potential mechanisms through which these molecules affect skeletal muscle is needed to optimize their use. The objective of this study is to synthesize preclinical, metabolomic, and clinical evidence on the effects of GLP-1RAs and dual/triple agonists on skeletal muscle, with a specific comparison between semaglutide and tirzepatide. This narrative review was based on a comprehensive literature search of PubMed/MEDLINE, Scopus, and Google Scholar, encompassing articles published through June 2026. This search was supplemented by manual citation tracking, which identified additional studies including preclinical and metabolomic investigations, randomized controlled trials, observational studies, and meta-analyses. Preclinical data suggest that GLP-1R/GIPR activation can positively modulate anabolic pathways, mitochondrial biogenesis, and muscle inflammation. Metabolomic evidence reveals lipid and amino acid remodeling compatible with improved mitochondrial function. Clinically, lean mass loss proportional to weight reduction is consistently observed, generally without meaningful declines in strength or performance; data for tirzepatide are more limited. Lean mass, however, is not synonymous with skeletal muscle. A paradox emerges between the presence of protective molecular signals and the clinical evidence of lean-mass loss. This phenomenon may be explained by a systemic energy/protein deficit rather than a direct catabolic effect of the drugs, although current evidence does not fully distinguish these mechanisms. The effectiveness of weight reduction should be considered a pivotal metric in clinical practice, particularly in populations susceptible to sarcopenia.

## 1. Introduction

Glucagon-like peptide-1 (GLP-1) and glucose-dependent insulinotropic polypeptide (GIP) are two incretin hormones that play a key role in postprandial metabolic regulation. Their characterization has led to an understanding of the so-called incretin effect, namely, the increased insulin secretion observed following oral glucose intake compared to intravenous administration of the same substrate [1,2]. This phenomenon has provided the physiological basis for the development of therapies capable of replicating or enhancing endogenous incretin signals [3,4].

GLP-1 is produced by L cells in the ileum and colon and is secreted in response to nutrients. It stimulates insulin secretion in a glucose-dependent manner, inhibits glucagon release, slows gastric emptying, and modulates food intake through mechanisms involving the central nervous system and satiety circuits [4,5]. GIP, the first incretin hormone to be identified, is secreted by K cells located in the duodenum and jejunum and plays a significant role in enhancing insulin secretion in the presence of glucose [1,3,4,6]. In addition to its insulinotropic effect, GIP appears to contribute to lipid metabolism and the regulation of adiposity, although these effects are more complex and less well-defined than those of GLP-1 [1,6].

**Figure 1:**
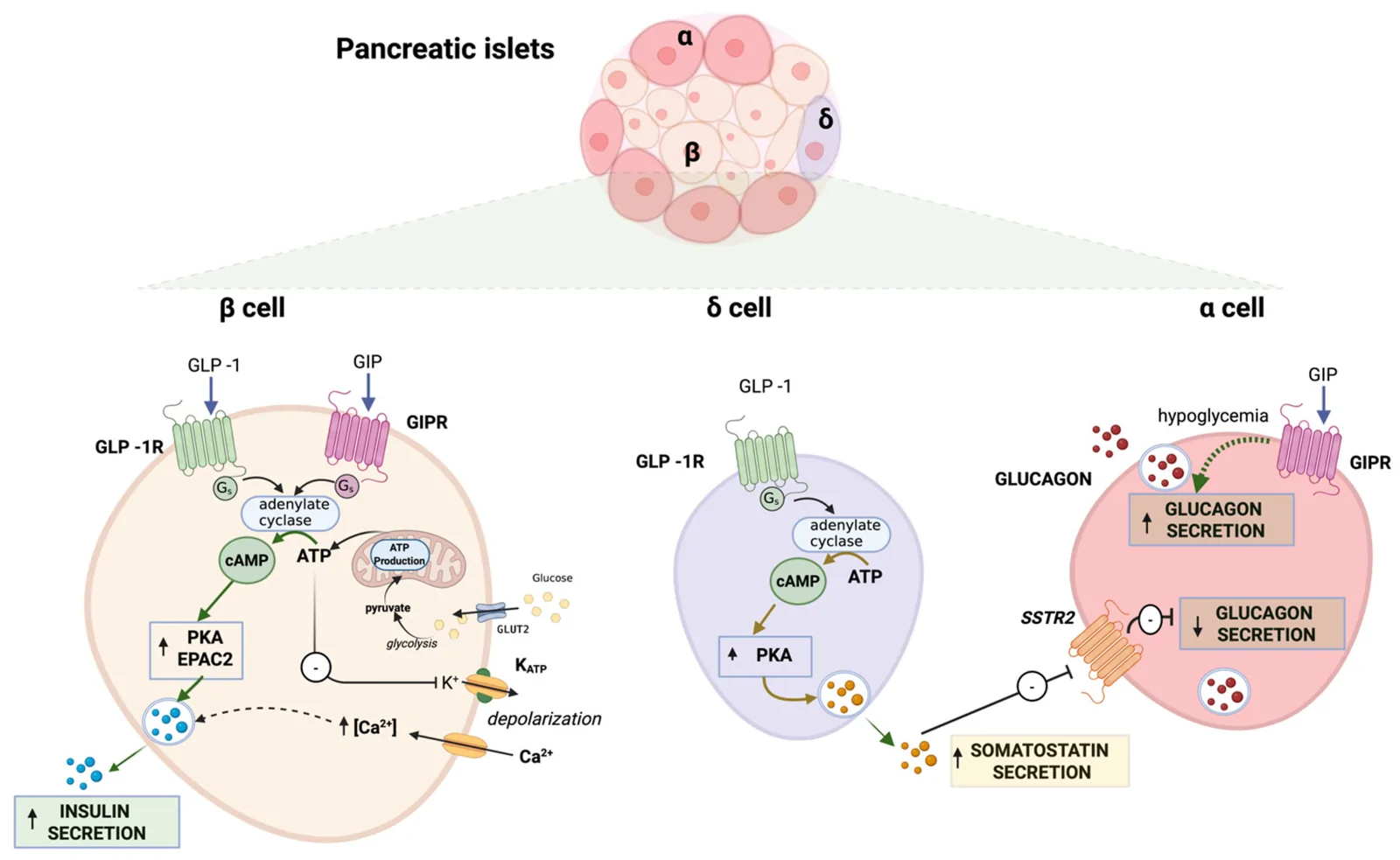
Signaling mediated by GLP-1 receptors (GLP-1R) and GIP receptors (GIPR) in β, δ, and α cells of the pancreatic islet. Schematic representation of the islets of Langerhans (**top**), consisting of β, α, and δ cells, and the signal transduction pathways activated in each cell type by incretin receptors. β-Cell (**left**). The binding of GLP-1 and GIP to their respective receptors (GLP-1R and GIPR) activates Gαs and adenylate cyclase, increasing cAMP concentration and leading to the activation of PKA and Epac2 [8]. Epac2 mediates, in part independently of PKA, the activation of Rap1 and the sensitization of intracellular Ca^2+^ release channels and exocytosis mechanisms [9]. This pathway converges with the glucose-dependent “trigger” pathway: glucose uptake via GLUT2, glycolysis, and mitochondrial ATP production induce the closure of K-ATP channels, membrane depolarization, the opening of voltage-dependent Ca^2+^ channels, and an increase in [Ca^2+^], which triggers insulin secretion. δ-Cell (**center**). The GLP-1R, coupled to Gαs, stimulates cAMP production and PKA activation, promoting somatostatin secretion [10]. α-Cell (**right**). Somatostatin released by the δ-cell acts paracrinally on the SSTR2 receptor of the α-cell, inhibiting glucagon secretion. Pharmacological blockade of SSTR2 eliminates the inhibitory effect of GLP-1 on glucagon secretion, demonstrating the indirect/paracrine nature of this inhibition [11]. GIP, via the GIPR, conversely exerts a stimulatory effect on glucagon secretion, particularly evident under conditions of hypoglycemia or euglycemia [12]. The figure does not include the emerging α → β paracrine pathway whereby α-cell-derived glucagon/GLP-1 activates GLP-1R on β-cells. This mechanism may contribute to, or complement, the classic incretin action of intestinal GLP-1, in addition to glucagon, which would act as an additional, albeit less potent, ligand. Abbreviations: Gs: stimulating G protein (Gαs subunit); AC: adenylate cyclase; cAMP: cyclic adenosine monophosphate (cAMP); PKA: protein kinase A; Epac2: exchange protein directly activated by cAMP 2 (cAMP-activated guanine nucleotide exchange factor, isoform 2); K-ATP: ATP-sensitive potassium channel; SSTR2: somatostatin receptor type 2. Graphic symbols: green arrow = activation/stimulation; “–” symbol (inhibition bar) = inhibition; dashed arrow = conditioned relationship (e.g., enhanced by hypoglycemia). Created in BioRender ©2026. Cautela, D. (2026) https://BioRender.com/y0n9cc0 (accessed on 5 August 2026).

Both hormones exerted their effects through binding to their respective receptors, GLP-1R and GIPR, which are highly expressed on pancreatic β-cells. Receptor activation stimulates intracellular signaling pathways that enhance pancreatic β-cell function and insulin secretion (Figure 1) [4]. Notably, the insulinotropic effects of both GLP-1 and GIP are glucose dependent, contributing to the low risk of hypoglycemia associated with incretin-based therapies when used as monotherapy or in combination with non-secretagogue medications [4,7].

In recent years, pharmacological interest in the incretin system has expanded substantially, leading to the development of GLP-1 receptor agonists (GLP-1RAs), and more recently, dual GLP-1/GIP agonists designed to harness the complementary metabolic actions of these pathways. Evidence that concomitant activation of GLP-1R and GIPR can produce synergistic metabolic responses has provided the rationale for the development of dual incretin agonists in the treatment of type 2 diabetes mellitus (T2DM) and obesity.

In addition to synthetic incretin-based therapies, natural products may provide a complementary perspective on the regulation of glucose metabolism. Polysaccharides derived from edible fungi have been reported to exert hypoglycemic effects through multiple mechanisms, including improvement in insulin sensitivity, modulation of gut microbiota, and regulation of intracellular signal-transduction pathways [13]. This evidence does not imply equivalence between natural products and approved GLP-1-based pharmacotherapies; rather, it highlights the broader biological context in which dietary and pharmacological interventions may influence glucose and energy metabolism.

GLP-1 and GIP are linear peptides derived from the proteolytic cleavage of the proglucagon and pro-GIP precursors, characterized by a specific amino acid sequence that allows them to bind to their respective G protein-coupled receptors (GPCRs) expressed on pancreatic β-cells [1,14,15]. GLP-1RAs are modified peptides analogues designed to enhance plasma stability, resist degradation by dipeptidyl peptidase-4 (DPP-4), and prolong their half-life (Table 1). Among these, liraglutide, dulaglutide, and semaglutide are some of the most clinically significant examples. Semaglutide, in particular, has become one of the most studied and widely used GLP-1RAs due to its weekly dosing regimen and marked efficacy in improving glycemic control and reducing body weight [16,17].

Pharmacological modifications include targeted amino acid substitutions, acylation, or conjugation with high-molecular-weight molecules, resulting in important pharmacokinetic properties among the available agents (Table 1). These modifications have been demonstrated to enhance plasma stability, thereby enabling weekly or daily administration. Examples include liraglutide, a C16-palmitoylated GLP-1 analogue that binds to serum albumin [18,19]. Dulaglutide is a GLP-1 analogue fused through a peptide linker to a modified IgG4 Fc fragment, thus increasing molecular size and reducing renal clearance [22,23]. Semaglutide contains Aib at position 8, Arg at position 34, and a C18 fatty diacid attached to Lys26 via a hydrophilic spacer; albumin binding prolongs its half-life to approximately one week [20,21].

These molecules maintain the three-dimensional conformation necessary to activate the GLP-1R, which triggers the intracellular signaling cascades mediated by adenylate cyclase, and in turn, enhances insulin secretion [4].

The development of dual GLP-1/GIP agonists, which combine the simultaneous activation of both incretin receptors, has introduced a new class of drugs with substantial efficacy for metabolic control and weight loss. This strategy is supported by evidence that GLP-1 and GIP exert complementary effects on the regulation of carbohydrate and lipid metabolism, and that their co-stimulation can produce more potent metabolic responses than activation of either receptor alone.

Tirzepatide, the leading representative of this class, is a 39-amino acid hybrid peptide with an acylated side chain that prolongs its half-life. It has been designed to simultaneously activate GLP-1R and GIPR, with synergistic effects on blood glucose, body weight, and various cardiometabolic parameters [15,24,25,28] (Table 1).

Clinical trials have demonstrated that tirzepatide elicits a more substantial reduction in body weight in comparison to other incretin-based medications. This finding suggests the emergence of novel therapeutic avenues for the management of obesity and T2DM [29].

The most recent evolution in this pharmacological approach is represented by the development of triple receptor agonists, which combine the simultaneous activation of GLP-1, GIP, and glucagon receptors. This class of molecules was designed to integrate the anorexigenic and insulinotropic effects of GLP-1 and GIP with the increase in energy expenditure mediated by the glucagon receptor activation. In preclinical models, triple agonists have demonstrated superior ability to reduce body weight and increase energy expenditure compared to mono- and dual-agonists, suggesting a potential pharmacological advantage in the management of obesity [30]. Among these compounds, retatrutide is currently the most advanced candidate in clinical development, with Phase 2 data demonstrating significant weight loss, improved glycemic control, and benefits across various cardiometabolic parameters, with an overall acceptable safety profile [26,27]. However, the potential effects of triple receptor agonism on skeletal muscle require specific consideration. Retatrutide, a triple GLP-1/GIP/glucagon receptor agonist, represents a significant pharmacological advancement. A phase 2 trial demonstrated that retatrutide resulted in significant weight loss over a period of 48 weeks. However, muscle-specific outcomes were not designated as primary endpoints. Consequently, the implications of its potential impact on weight reduction, muscle quantity, quality, and function remain unresolved [26].

The capacity of these agents to induce substantial and sustained weight loss—surpassing that attainable with conventional therapies—led to their increasing widespread use, even in non-diabetic subjects and across a broad range of clinical settings [31,32,33]. This growing use has shifted scientific attention beyond their conventional metabolic effects toward their influence on body composition, particularly lean mass and skeletal muscle health [31,32,33].

These observations have raised concerns, particularly for older adults, subjects with sarcopenic obesity, and those with an already reduced muscle reserve [34,35]. Skeletal muscle is a fundamental determinant of metabolic health, and beyond serving as the primary site of insulin-mediated glucose uptake, plays a pivotal role in regulating basal metabolism, thermogenesis, immune function, and the production of myokines with systemic endocrine effects [36]. A reduction in muscle mass has been shown to be associated with worsened insulin sensitivity, an increased risk of frailty, functional decline, and higher mortality, especially in older adults or those with metabolic comorbidities [34]. Therefore, understanding how treatments for obesity and T2DM influence skeletal muscle is essential for evaluating the efficacy on weight loss and its long-term health implications.

A substantial body of clinical research has documented that a considerable proportion of the weight reduction induced by GLP-1RAs is attributable to a decrease in lean mass, with percentages ranging from 20% to 40% of total weight loss [37,38,39]. Although a reduction in lean mass is generally expected during weight loss, the extent of this phenomenon observed in certain trials has drawn concerns about potential sarcopenia-related risk, particularly in more vulnerable populations such as the elderly, individuals with sarcopenic obesity, or patients with metabolic frailty [34,35,40]. Indeed, the loss of lean mass is not merely a quantitative phenomenon; it can result in reductions in strength, physical performance, and metabolic resilience, with significant clinical implications [41,42]. Importantly, lean mass (or lean soft tissue/fat-free mass, depending on the assessment method) is not synonymous with skeletal muscle mass and includes non-muscle components and fluid-related variation. Therefore, reductions in lean mass should not be equated with skeletal-muscle loss or sarcopenia without direct measures of muscle quantity or quality, strength, and physical performance [31].

However, the biological picture appears to be more complex than what clinical data alone suggest. Preclinical studies have indicated that GLP-1R activation has the potential to exert a positive modulatory effect on anabolic pathways, enhance mitochondrial biogenesis, reduce systemic inflammation, and promote muscle function [43]. Tirzepatide, through its dual activation of GLP-1R and GIP-R, may elicit distinct effects on muscle metabolism compared to conventional GLP-1RAs. However, the extant evidence remains preliminary and has not yet been integrated into a coherent synthesis [15]. This phenomenon gives rise to an apparent paradox: molecular signals that potentially offer protection appear to coincide with a clinical loss of lean mass that in some cases, may be clinically significant.

The analysis of metabolomic data reveals alterations in amino acid profiles and markers of protein turnover during treatment with GLP-1RAs, thereby suggesting a direct involvement of muscle and mitochondrial metabolism [44,45]. Notwithstanding the value of this evidence, a unified interpretive framework integrating molecular dynamics, metabolic signals, and clinical phenotypes has yet to be established.

In light of these considerations, this review aims to synthesize the preclinical, metabolomic, and clinical evidence regarding the effects of GLP-1RAs, dual GLP-1/GIP agonists, and the most recent triple GLP-1/GIP/glucagon agonists on skeletal muscle, with a comparative focus on semaglutide and tirzepatide. In particular, the objectives are to elucidate the molecular mechanisms involved; assess the significance of the loss of lean mass observed in clinical trials; and discuss the potential implications for clinical practice, with a particular focus on individuals most susceptible to the development of sarcopenia.

## 2. Search Strategy and Selection Criteria

This study was conducted as a narrative review of the literature, with the aim of synthesizing the currently available evidence regarding the relationship between the use of GLP-1 RAs and the risk of sarcopenia.

The choice of a narrative design, rather than a systematic one, was motivated by the heterogeneity of the literature on this topic, which encompasses randomized clinical trials, post hoc and exploratory analyses, mechanistic studies in vitro and in animal models, as well as systematic reviews and consensus documents. A narrative approach therefore enables the integration of clinical evidence regarding the effects of GLP-1 RAs on body composition with physiological and molecular biology data concerning the mechanisms regulating muscle mass, providing a broader interpretive framework for integrating evidence across different levels of investigation than would be possible within a conventional systematic review.

Despite the absence of a formal definition of a PICO (population, intervention, comparison, outcome) question, the search strategy was guided by the four conceptual elements characteristic of this format, adapted to the narrative nature of our review: Population/Problem (adults, with particular attention to the elderly population and patients with T2DM and/or obesity, at risk for sarcopenia or diabetic sarcopenia); Intervention/Exposure (treatment with GLP-1 RAs, GIP/GLP-1 and GLP-1/GIP/glucagon co-agonists, such as liraglutide, dulaglutide, semaglutide, tirzepatide, and retatrutide); Comparison (placebo, other hypoglycemic agents, or no pharmacological intervention, as well as comparisons among different molecules within the class); and Outcome (changes in lean body mass, skeletal muscle mass, and muscle function; incidence or risk of sarcopenia and sarcopenic obesity). Although this conceptual framework did not constitute a formal review protocol, it guided the development of the search strings and the selection of the sources described below.

The literature search was conducted by consulting the electronic databases PubMed/MEDLINE, Scopus, and Google Scholar (as a supplementary tool to broaden the search), accompanied by a manual review of the bibliographic lists (“citation tracking”) of the most relevant articles, including articles published up to June 2026, in order to identify additional relevant sources not captured by the primary electronic search. We also consulted the Prescribing Information issued by the U.S. Food and Drug Administration for the main drugs in this class, as a regulatory source complementary to the peer-reviewed scientific literature.

No lower publication date restriction was applied to include historically foundational mechanistic reference studies. Combinations of keywords and MeSH terms related to GLP-1 receptor agonists, skeletal muscle, body composition, lean body mass, sarcopenia, metabolomics and the main drugs of interest—particularly semaglutide and tirzepatide—were used. The following terms were used in the search strategy applied to PubMed/MEDLINE as an illustrative example: (“GLP-1 receptor agonist” OR semaglutide OR tirzepatide OR liraglutide OR dulaglutide OR retatrutide) AND (“skeletal muscle” OR “lean mass” OR “lean body mass” OR sarcopenia OR “muscle strength” OR “body composition” OR metabolomics). Analogous search strings, adapted to the syntax of each database, were utilized in Scopus and Google Scholar.

Eligible articles were those published in peer-reviewed journals that investigated the effects of GLP-1RAs or GLP-1/GIP dual agonists on skeletal muscle, body composition, or muscle metabolism-related biomarkers. Preclinical studies, metabolomic studies, randomized clinical trials, observational studies and meta-analyses were included when they provided relevant data on molecular mechanisms or clinical effects on muscle. Conference abstracts lacking complete data, articles not available in full text, and studies irrelevant to muscle were excluded.

The selection process for articles was conducted in two phases. In the initial screening phase, the titles and abstracts of articles were reviewed to exclude those that were clearly irrelevant to the review question. In the second phase, the remaining articles were subjected to a full-text evaluation. At the conclusion of this phase, the final selection of included sources was determined. In accordance with the review’s narrative character, the selection placed greater emphasis on thematic pertinence and the caliber of the evidence, as opposed to adhering to a predetermined and documented systematic protocol [46]. Due to the multifaceted nature of the topic, we incorporated evidence from various sources. Preclinical studies were useful for elucidating molecular pathways and cellular mechanisms; metabolomic studies were essential for interpreting changes in amino acid, lipid, and mitochondrial profiles, and randomized clinical trials provided body composition measurements via dual-energy X-ray absorptiometry (DXA), bioelectrical impedance analysis (BIA), or computed tomography (CT). Because these methods quantify different compartments, DXA- or BIA-derived lean mass and CT-derived skeletal-muscle measurements were not considered interchangeable. Observational studies provided a real-world perspective, while previous meta-analyses offered contextual support. This integrative approach allowed for the construction of a coherent synthesis of heterogeneous evidence, as recommended for reviews of complex, multidisciplinary topics [46].

The information extracted from the included articles was organized according to a conceptual framework that begins with the pharmacology and mechanisms of action of GLP-1 RAs, moves on to their clinical efficacy on body composition, and concludes with a critical discussion of the risk of reduced muscle mass and function, with particular attention to the elderly population and individuals with sarcopenic obesity or diabetic sarcopenia. The evidence thus gathered was subsequently synthesized in narrative form, integrating the results of clinical trials with preclinical and mechanistic data. This synthesis was undertaken to provide an up-to-date and critically reasoned overview of the state of the art on the subject.

As narrative reviews do not aim to formally assess the strength of evidence, the level of investigation underlying each mechanism discussed in Section 3 is explicitly indicated throughout the text to assist readers in evaluating the reliability of the conclusions. Briefly, the GLP-1R-mediated GLUT4 translocation and glycogen-synthesis pathway is primarily supported by in vitro human muscle-cell and rodent skeletal-muscle models [47,48,49], whereas direct evidence of functional GLP-1R signaling in intact human skeletal muscle remains comparatively limited. The mTORC1/AMPK anabolic-signaling hypothesis and the mitochondrial-biogenesis hypothesis are largely derived from rodent models [50,51] and myotube/cell-culture models [50,52]. Human confirmation is currently limited to indirect exploratory metabolomic and body-composition substudies [53,54]. Evidence for the anti-inflammatory effects of GLP-1RAs is predominantly derived from narrative and mechanistic reviews rather than from primary human skeletal-muscle data [55,56]. Finally, the comparative pharmacology of semaglutide versus tirzepatide is based on indirect, cross-trial pharmacological reasoning [57,58] rather than on head-to-head randomized evidence with muscle-related endpoints. This hierarchy of evidence is reiterated at the relevant points throughout Section 3.

## 3. Molecular Mechanisms Mediating the Effect of GLP-1 RA on Skeletal Muscle

The effects of incretin therapies on skeletal muscle result from a complex interaction among the pharmacological properties of the drugs, receptor activation, intracellular signaling pathways, and the resulting clinical outcomes.

A comprehensive understanding of the effects of GLP-1RAs and GLP-1/GIP dual agonists on skeletal muscle necessitates a multilevel analysis that integrates preclinical, molecular, and physiological data. While most of the clinical attention has been directed toward the effects of these drugs on body weight and glucose metabolism, a mounting body of evidence suggests that the activation of incretin receptors exerts both direct and indirect influences on skeletal muscle by modulating anabolic, catabolic, mitochondrial, and inflammatory pathways.

Katsanos et al. (2026) [59] highlighted that GLP-1RAs enhance microvascular perfusion in muscle, thereby improving the delivery of insulin, glucose, and amino acids, and facilitating carbohydrate uptake and protein synthesis when plasma nutrient availability is high. However, during prolonged treatment, the energy deficit—and occasionally the protein deficit—induced by reduced food intake is associated with a loss of lean mass. As a result, the net effect on muscle protein turnover remains uncertain, underscoring the necessity for studies that explicitly control energy and protein intake to distinguish direct drug effects from those driven by nutritional restriction.

As thoroughly examined by Müller et al. (2019) [60], GLP-1 exerts a multitude of extra pancreatic effects that extend beyond its well-established incretin action. This section aims to provide a concise summary of the molecular mechanisms pertinent to skeletal muscle, with a particular focus on potential distinctions between semaglutide, other GLP-1 receptor agonists (GLP-1RAs), and tirzepatide.

### 3.1. Direct Effects of GLP-1 on Skeletal Muscle: Receptor Expression and Glucose Uptake

The GLP-1 receptor (GLP-1R) has traditionally been considered to be expressed primarily in the pancreas, intestine, and central nervous system [1]. However, subsequent studies have also identified the presence of GLP-1R in skeletal muscle, although expression levels vary and are not uniformly confirmed across species and experimental models [44,47,57]. The activation of this receptor in myocytes has been demonstrated to modulate critical processes involved in muscle homeostasis, including glucose uptake, insulin sensitivity, and protein turnover.

This observation remains a matter of debate. While studies conducted on mouse models suggest that GLP-1 may exert direct beneficial effects on skeletal muscle and bone tissue, there is a paucity of robust data in humans unequivocally confirming this direct action. A considerable body of research suggests that considering the minimal and variable expression of GLP-1R in human skeletal muscle, the clinically observed effects are principally indirect. These effects are mediated by systemic mechanisms, such as enhanced insulin sensitivity, diminished inflammation, and weight reduction, rather than by direct autocrine/paracrine action on myocytes.

**Figure 2:**
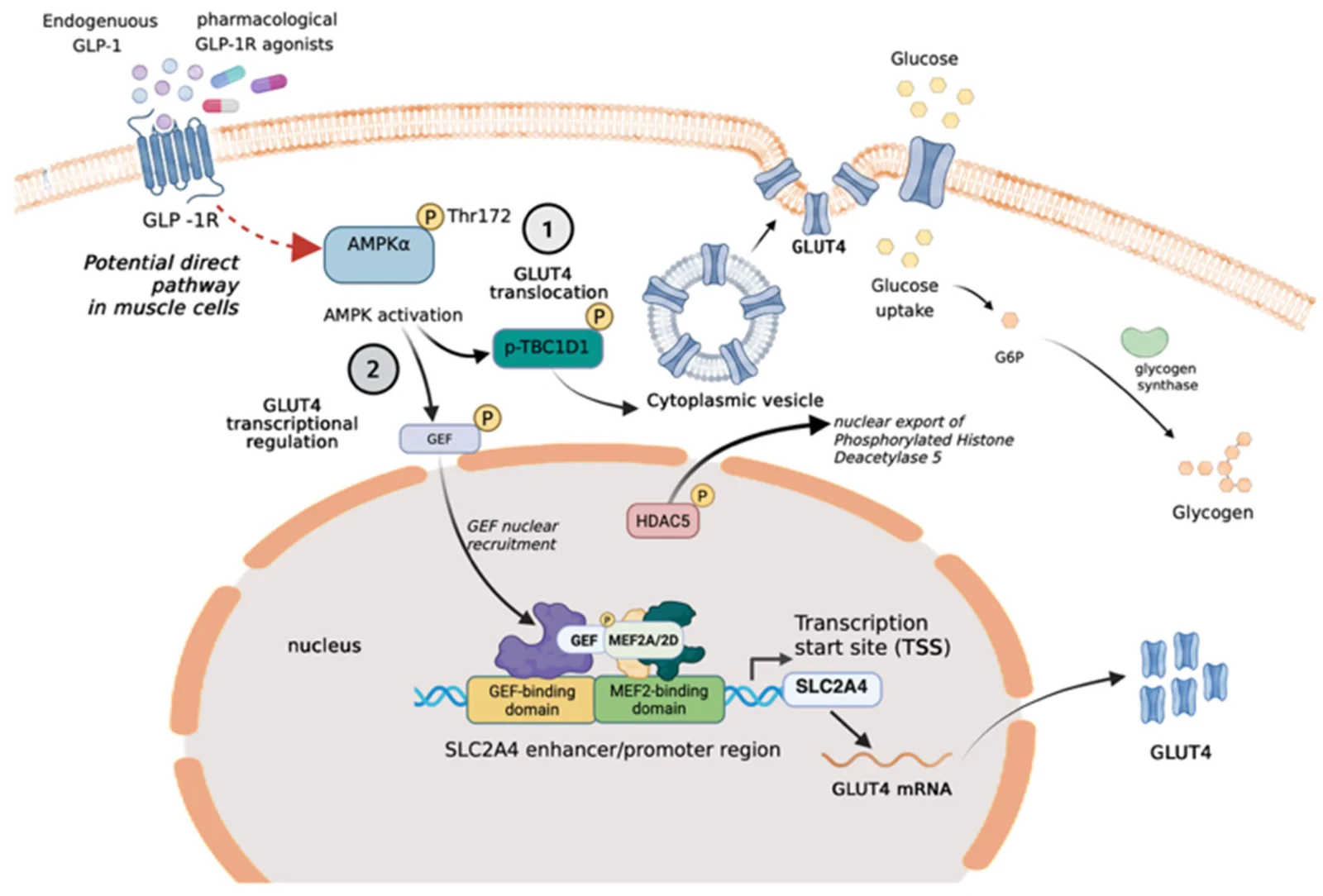
Proposed molecular mechanism for the direct regulation of glucose metabolism mediated by GLP-1R in skeletal muscle. Stimulation of the glucagon-like peptide-1 receptor (GLP-1R) by endogenous ligands or pharmacological agonists is proposed to promote the phosphorylation of AMP-activated protein kinase (AMPK) at threonine 172 (Thr172), although this direct pathway remains to be fully validated in this tissue [49]. AMPK activation triggers two distinct but converging pathways that promote glucose homeostasis. (1) GLUT4 vesicle translocation: GLP-1R-induced activation of AMPK leads to the phosphorylation of TBC1D1, promoting the translocation of GLUT4 transporters from cytoplasmic vesicles to the plasma membrane. This is followed by the influx of blood glucose, which is subsequently converted to glucose-6-phosphate (G6P) and incorporated into glycogen via the enzyme glycogen synthase. (2) Transcriptional regulation of the SLC2A4 gene: AMPK promotes the phosphorylation of the GLUT4 enhancer factor (GEF) and its subsequent recruitment to the nucleus [13]. Concurrently, the phosphorylated histone deacetylase 5 (p-HDAC5) is exported from the nucleus. In the nucleus, the GEF complex binds to the specific domain on the SLC2A4 gene enhancer/promoter (transcription start site, TSS) by interacting with the MEF2A/2D factors [52]. This drives the expression of GLUT4 mRNA and the subsequent synthesis of new GLUT4 proteins. Created in BioRender. Cautela, D. (2026) https://BioRender.com/y0n9cc0 (accessed on 5 August 2026).

GLP-1 activity, through endogenous or exogenous GLP-1 or GLP-1R agonists, has been demonstrated to increase glycogen synthesis and glucose uptake in skeletal muscle by activating AMPK [49]. Figure 2 illustrates the proposed molecular mechanism underlying the direct regulation of glucose metabolism in skeletal muscle mediated by GLP-1R, highlighting the activation of AMP-activated protein kinase (AMPK) and the pivotal transcriptional regulators involved in GLUT4 expression and glucose homeostasis.

Wu et al. (2022) [49] demonstrated that AMPK activation enhances the phosphorylation of the GLUT4 enhancer factor (GEF), a transcription factor that interacts with myocyte enhancer factor 2 (MEF2) to induce GLUT4 gene (Gene: SLC2A4) expression.

A comprehensive analysis of the human GLUT4 promoter has identified a region of approximately 2.4 kilobases (kb) upstream of the transcription start site that contains the elements necessary for proper, tissue-specific, regulation of the gene in response to hormonal and metabolic changes, such as those induced by fasting [48]. Deletion studies have identified a 895-base pair region as functionally essential, comprising two highly conserved domains [61]. The first is capable of binding GEF, while the second domain contains a binding site for MEF2. MEF2A and MEF2D bind to the regulatory element in the GLUT4 promoter as functional dimers and contribute to the hormonal regulation of GLUT4 expression [62]. The regulation of GLUT4 does not depend on a solitary factor, but rather on a collaborative network. Indeed, GEF and MEF2A, when expressed individually, induce only a modest activation of the GLUT4 promoter. On the other hand, their combined expression leads to a substantial augmentation in promoter activity, reaching a 4–5-fold increase [62]. Indeed, both MEF2 binding site and domain 1 of GLUT4 promoter, in conjunction with their respective binding factors, are indispensable for complete GLUT4 expression in skeletal muscle.

Additional factors, including Myogenic Differentiation Factor (MyoD), Thyroid Hormone Receptor Alpha (TRα), Krüppel-Like Factor 15 (KLF15), and Peroxisome Proliferator-Activated Receptor Gamma Coactivator 1 Alpha (PGC-1α), have been shown to enhance MEF2 activity [63,64,65,66]. Nuclear Respiratory Factor 1 (NRF-1), a target of PGC-1α, has been demonstrated to increase both GLUT4 expression and muscle oxidative capacity [63,65], thereby establishing a link between muscle oxidative capacity and GLUT4 expression.

MEF2 is also subject to repression by Class II histone deacetylases (HDACs), particularly HDAC4, HDAC5, HDAC7, and HDAC9. The repressive activity of these HDACs is removed through phosphorylation and subsequent nuclear export or proteasomal degradation. These processes are regulated by kinases such as Calcium/Calmodulin-Dependent Protein Kinase (CaMK) and AMP-Activated Protein Kinase (AMPK). Both kinases are capable of increasing GLUT4 expression through the phosphorylation of HDAC5 and the activation of MEF2 [67,68].

Calcineurin, a calcium-dependent phosphatase, activates MEF2 either directly or via the Nuclear Factor of Activated T Cells (NFAT), thereby also contributing to increased GLUT4 expression, with a predominant role in type II muscle and a redundant role with CaMK and AMPK in type I muscle [69,70].

In conclusion, GLUT4 abundance is regulated not only at a transcriptional level but also subject to protein turnover, since the transporter undergoes degradation via Calpain 2 action [71]. Furthermore, the expression of Calpastatin, a naturally occurring inhibitor of Calpain 2, has been observed to increase steady-state GLUT4 levels, thus delineating a multilevel system in which metabolic, hormonal, contractile, and epigenetic signals converge to finely regulate the expression of the transporter in skeletal muscle.

### 3.2. GLP-1 Signaling Mediated by Non-Canonical Receptors and via GPI/IPG

**Figure 3:**
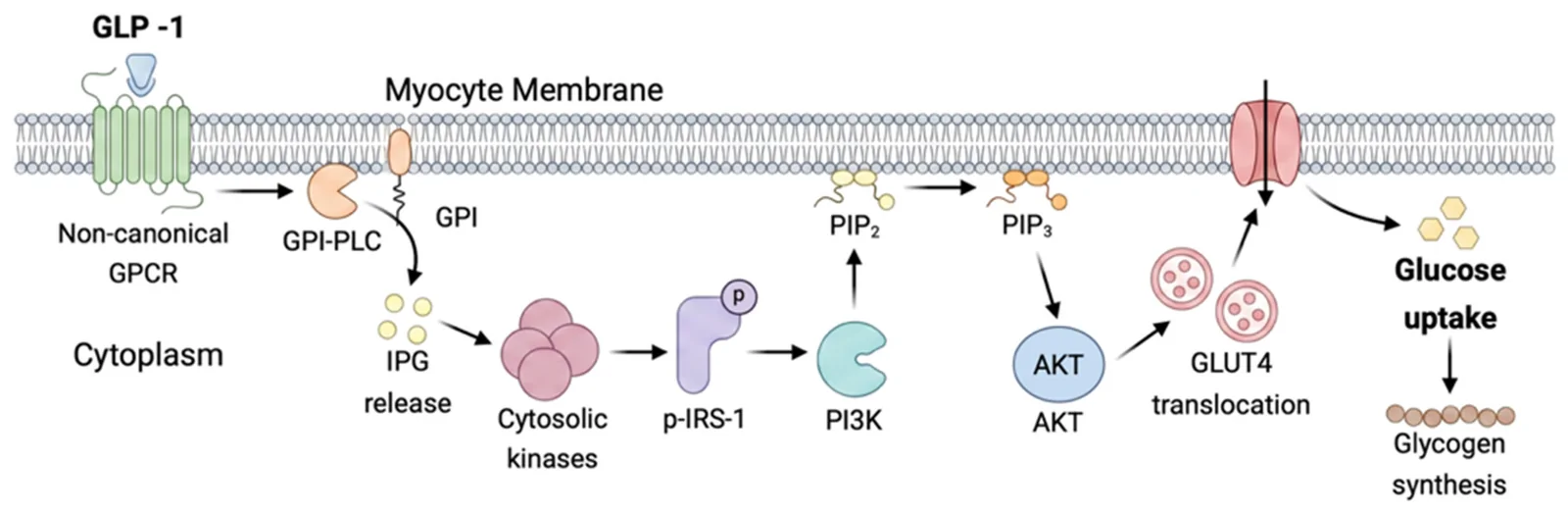
Proposed non-canonical GLP-1 signaling pathway in skeletal muscle. GLP-1R belongs to the G protein-coupled receptor (GPCR) superfamily; however, GLP-1 does not significantly increase cAMP in myocytes, raising the possibility that GLP-1 action in skeletal muscle is mediated through signaling mechanisms distinct from those of the classical pancreatic GLP-1R [62]. According to this proposed mechanism, GLP-1 binds to a putative non-canonical receptor on the myocyte membrane, activating a membrane phospholipase (GPI-PLC) that hydrolyzes glycosylphosphatidylinositols (GPIs) and releases inositol phosphoglycans (IPGs) into the cytoplasm as intracellular second messengers [63]. IPGs are proposed to recruit and activate cytosolic kinases that phosphorylate Insulin Receptor Substrate 1 (IRS-1); phosphorylated IRS-1 would then bind and activate phosphatidylinositol 3-kinase (PI3K), which translocates to the plasma membrane and converts phosphatidylinositol 4,5-bisphosphate (PIP_2_) to phosphatidylinositol 3,4,5-trisphosphate (PIP_3_). The resulting increase in PIP_3_ promotes membrane recruitment and the activation of protein kinase B (AKT), triggering an insulin-like signaling cascade that drives GLUT4 translocation to the plasma membrane, increased glucose uptake, and glycogen synthesis. This proposed IPG-to-IRS-1 link has not been directly demonstrated and represents an inferential extension bridging documented IPG generation and PI3K/AKT activation by GLP-1 in human myocytes [63]. Notably, chronic hyperglycemia has been shown to attenuate GLP-1-induced GLUT4 translocation in human skeletal muscle cells, suggesting that this pathway may be functionally impaired under conditions of sustained hyperglycemia [47]. Created in BioRender. Cautela, D. (2026) https://BioRender.com/y0n9cc0 (accessed on 5 August 2026).

GLP-1R belongs to the G protein-coupled receptor (GPCR) superfamily, but GLP-1 does not significantly increase cAMP in myocytes. This has raised the possibility that GLP-1 action in skeletal muscle may be mediated through signaling mechanisms different from those of the classical GLP-1R (Figure 3). In this regard, studies on extra-pancreatic cells have identified alternative GLP-1 binding sites capable of activating tyrosine kinase pathways and unconventional G proteins. Accordingly, Montrose-Rafizadeh et al. (1997) demonstrated that GLP-1 can also activate cAMP-independent signaling pathways in adipocytes, suggesting that GLP-1 signaling may occur through receptors or signaling mechanisms distinct from the canonical pancreatic GLP-1R [72].

The study conducted by Luque et al. (2002) [73] thoroughly examined the impact of GLP-1 on glucose metabolism in human skeletal muscle. The researchers observed that the hormone’s signal is transmitted through the hydrolysis of glycosylphosphatidylinositols (GPI), a process that leads to the formation of inositol phosphoglycans (IPG), which function as intracellular second messengers. These effects occur at hormone concentrations that are normally found in the human body, suggesting that GLP-1 acts as a natural, daily regulator of blood sugar levels, optimizing glucose uptake specifically through the muscle compartment.

Experimental evidence suggests that in extra-pancreatic tissues, GLP-1 may activate an alternative signaling pathway independent of the canonical GLP-1 receptor. According to this proposed mechanism, GLP-1 binding to a putative non-canonical receptor activates a membrane phospholipase, which hydrolyzes glycosylphosphatidylinositols (GPIs) and releases inositol phosphoglycans (IPGs) into the cytoplasm. These secondo messengers recruit and activate cytosolic kinases, leading to the phosphorylation of Insulin Receptor Substrate 1 (IRS-1). Phosphorylated IRS-1 binds to and activates phosphatidylinositol 3-kinase (PI3K), which translocates from the cytoplasm to the inner surface of the cell membrane and catalyzes the phosphorylation of phosphatidylinositol 4,5-bisphosphate (PIP_2_) to generate phosphatidylinositol 3,4,5-trisphosphate (PIP_3_). The consequent increase in PIP3 levels promote the membrane recruitment and activation of protein kinase B (AKT), thereby prompting an insulin like signaling cascade, with GLUT4 translocation to the plasma membrane and increased glucose uptake and glycogen synthesis. Nevertheless, Green et al. (2012) [47] demonstrate that chronic hyperglycemia induces GLP-1 resistance in skeletal muscle. The proposed mechanism does not appear to block the initial signaling; rather, it may attenuate GLUT4 translocation, thereby hindering glucose uptake, and consequently, glycogen synthesis, even in the presence of the GLP-1 [47].

### 3.3. Anabolic Pathways: mTORC1, AMPK, and eEF2

mTOR has been identified as a critical regulator of the processes involved in mRNA translation and protein synthesis. mTOR exists in two functionally distinct complexes, mTOR complex 1 (mTORC1) and mTOR complex 2 (mTORC2). mTORC1 primarily regulates anabolic processes, including protein synthesis and cell growth, whereas mTORC2 contributes to cell survival, metabolism, and cytoskeletal organization. Upon activation, mTORC1 has been shown to promote cell growth by stimulating protein synthesis through the phosphorylation of several downstream targets, most notably the 70-kDa ribosomal S6 kinase (p70S6K1) and the eukaryotic initiation factor 4E-binding protein 1 (4E-BP1). Recent evidence suggests that mTORC1 and AMPK act as two opposing forces that regulate muscle adaptation to nutrition, starvation, and growth stimuli [74,75,76].

As demonstrated in several studies, GLP-1 and its agonists significantly modulate muscle metabolism via the activation of AMPK [76]. AMPK antagonizes mTORC1 by exerting an inhibitory effect on protein synthesis in cultured muscle cells. This antagonism occurs through the simultaneous repression of mTOR signaling and the activation of MAPK signaling. The combined actions of these two effects regulate the initiation and elongation phases of mRNA translation [77].

In skeletal muscle, the action of AMPK on the mTORC1 complex is now understood as a multilevel inhibitory mechanism: AMPK has been shown to phosphorylate mTOR at Thr2446, thereby reducing the ability of the mTORC1 complex to activate in response to anabolic signals [78]. This phosphorylation hinders that of the nearby Ser2448 site, which was traditionally considered a marker of mTORC1 activation. Nevertheless, recent studies suggest that its functional significance may depend on cellular context and metabolic condition, and that it may also be considered as a predominantly inhibitory signal under conditions of energy stress or AMPK activation [79].

Emerging evidence indicates that both sites, Thr2446 and Ser2448, contribute to the suppression of mTORC1 activity when phosphorylated, acting as a molecular brake that limits protein synthesis and cell growth under conditions of energy deficiency, thereby allowing AMPK to finely coordinate energy metabolism with muscle growth and adaptation processes.

In addition to its inhibitory effect on mTORC1, AMPK also regulates protein synthesis by inhibiting the activity of eukaryotic elongation factor 2 (eEF2). It has been shown that eEF2phosphorylation at Thr56 inhibits its binding with the ribosome, thereby slowing the rate of elongation. The phosphorylation of eEF2 at this site is catalyzed by eEF2 kinase (eEF2K). AMPK influences eEF2K activity through two distinct mechanisms [80].

The first pathway involves p70S6K, which acts downstream of mTOR. p70S6K phosphorylates and inhibits eEF2K, thereby activating eEF2. AMPK can counteract this effect by inhibiting the mTOR pathway. On the other hand, AMPK directly phosphorylates and activates eEF2K, leading to the inactivation of eEF2.

Although these mechanisms are well-documented in cellular models and in various tissues, the true extent of the AMPK-mediated regulation of eEF2 in skeletal muscle in vivo remains poorly defined. The available evidence suggests a potentially significant role, but specific studies are needed to clarify the quantitative impact of this axis on muscle protein synthesis under physiological and pathological conditions.

In addition to its effects on mTORC1 and eEF2, GLP-1 has also been shown to promote glycogen anabolism. GLP-1, whether endogenous or exogenous, and GLP-1R agonists have been observed to enhance glucose uptake in skeletal muscle via the activation of AMPK. Furthermore, GLP-1 has been demonstrated to increase glycogen synthesis [49].

A study on cultured human myocytes revealed that GLP-1 mediates an increase in glycogen synthase-α activity [49]. Exendin-4, a GLP-1 receptor agonist, led to dose-dependent increases in both glucose uptake and glycogen synthase-α activity. In addition, Exendine-4 significantly promoted glucose uptake, type I fiber formation, and mitochondrial respiration.

### 3.4. Mitochondrial Metabolism and Bioenergetics

GLP-1R activation in murine skeletal muscle increased glycogen storage, glucose uptake, and mitochondrial biogenesis. Two studies have identified the GLP-1R/AMPK axis as a key mechanism through which incretin signaling modulates mitochondrial function and metabolic remodeling in skeletal muscle [49,51]. One study defined the direct molecular mechanism, while the other documented its physiological effects in vivo in a mouse model. AMPK acts as a cellular energy sensor, and once phosphorylated, activates a transcriptional cascade that converges on PGC-1α, considered the primary coactivator of mitochondrial biogenesis [50,52].

A comprehensive review of the scientific literature indicates that therapy with GLP-1RAs exerts a beneficial therapeutic effect on the ultrastructural properties of mitochondria within skeletal muscle tissue [34]. This effect is demonstrated by a statistically significant increase in parameters related to total mitochondrial area and organelle number, as well as a marked improvement in cellular morphology and structural integrity. At the molecular level, the action of these drugs results in the stimulation of de novo mitochondrial biogenesis, which is mediated by the activation of highly conserved intracellular signaling pathways, notably AMPK/SIRT1/PGC-1α. This offers a potential mechanism to explain their ability to counteract muscle atrophy and cellular senescence processes induced by metabolic insults [44,45].

Further trials are needed to determine whether these changes translate into clinical benefits related to muscle strength and physical performance.

### 3.5. Role of Inflammation and Oxidative Stress

Low-grade chronic inflammation, a hallmark of obesity, plays a pivotal role in the development of muscle dysfunction and sarcopenia. As substantiated by findings in both preclinical models and clinical studies conducted on patients diagnosed with obesity and T2DM, GLP-1RAs elicit both systemic and local anti-inflammatory effects. These pharmaceutical agents have been demonstrated to curtail the expression and secretion of pro-inflammatory cytokines, including TNF-α, IL-6, and MCP-1, by impeding signaling pathways such as that mediated by NF-κB, attenuating oxidative stress, and enhancing insulin sensitivity. These effects occur both systemically and in key tissue compartments involved in metabolic inflammation (adipose tissue, vascular wall, articular cartilage), contributing to a reduction in cardiovascular risk and a favorable modulation of the inflammatory microenvironment [55,56].

The reduction in oxidative stress, mediated by improved mitochondrial function, represents an additional mechanism through which GLP-1RAs can preserve muscle tissue integrity [44,45]. These anti-inflammatory and antioxidant effects may help in preserving muscle function, even in the presence of a reduction in lean mass.

### 3.6. Potential Differences Between Semaglutide, Other GLP-1RAs, and Tirzepatide

The dual activation of GLP-1R and GIPR by tirzepatide introduces additional biological complexities. GIPR is expressed in skeletal muscle, and its activation has been shown to increase glucose uptake, stimulate protein synthesis via Akt/mTOR, and improve mitochondrial function [58]. This finding suggests the possibility of distinct muscular effects of tirzepatide compared to pure GLP-1RAs.

A broad overview of the extant preclinical evidence indicates that GLP-1RAs and dual agonists may offer a promising therapeutic potential for the protection of skeletal muscle. This potential is evidenced by improvements in metabolic quality, mitochondrial function, and inflammatory profile. However, the clinically observed loss of lean mass may be driven primarily by systemic factors related to weight loss, including energy deficit, reduced protein intake, and lifestyle changes, rather than by a direct catabolic effect of the drugs; however, weight-loss-matched comparative data are scarce.

Retatrutide further extends this pharmacological continuum by adding glucagon-receptor agonism to the dual GIP/GLP-1 activity of tirzepatide. Glucagon-receptor signaling has been demonstrated to increase hepatic energy expenditure and fatty-acid oxidation [26]. This mechanism is theorized to favor fat oxidation over amino-acid catabolism. In the phase 2 substudy conducted in patients with T2DM, the proportion of lean mass loss relative to total weight loss with retatrutide was comparable to that observed with other high-efficacy incretin therapies [81], providing reassurance that greater efficacy did not translate into disproportionate muscle loss in that specific population.

However, it should be noted that the magnitude of weight loss in this substudy was substantially lower than that observed with Retatrutide in the obesity population (16.9% vs. up to 24.2%), and no equivalent body-composition data are available at that higher magnitude. In light of the theoretical discordance between glucagon-driven amino acid consumption and fat oxidation sparing, the net effect of Retatrutide on skeletal muscle remains an open question. Dedicated body-composition and functional studies in ongoing phase 3 trials are necessary to elucidate this effect.

## 4. Metabolomic Evidence: Integrating Metabolomic Signatures with Molecular Mechanisms and Clinical Muscle Outcomes

The field of metabolomics has made an essential contribution to the understanding of the effects of GLP-1RAs and GLP-1/GIP dual agonists on skeletal muscle. A comprehensive analysis of amino acid, lipid, and mitochondrial profiles facilitates the identification of systemic changes that reflect protein balance, mitochondrial function, and muscle turnover. The available data demonstrate a complex metabolic remodeling process that integrates signals promoting muscle quality with physiological adaptations to the treatment-induced energy deficit.

GLP-1RAs, including liraglutide, dulaglutide, and semaglutide, have been shown to induce systemic remodeling of the circulating metabolome, characterized by coordinated alterations in lipids, amino acids, and energy metabolites. The observed modifications are consistent with improved insulin sensitivity, mitochondrial function, and a reduction in lipotoxic and inflammatory burden [54,82].

These metabolomic findings may provide a link between the molecular mechanisms described above and the clinical changes in body composition; however, their interpretation requires caution.

One of the most consistent metabolomic patterns observed with GLP-1RAs involves the remodeling of plasma lipidome. In patients with T2DM and subclinical cardiac dysfunction, 18 weeks of liraglutide treatment, compared with glimepiride, resulted in a significant reduction in several biological markers, including ceramides, sphingomyelins, lysophosphatidylcholines, cholesterol esters, and alkyl-ether-structured phosphatidylcholines, despite comparable glycemic control. These lipidomic changes are consistent with a reduction in lipotoxicity and cardiovascular risk [54]. Ceramides and other sphingolipids are recognized mediators of insulin resistance, cellular apoptosis, and endothelial dysfunction. In addition, a modulation of bile acid pathways and arachidonic acid metabolism is observed, with anti-inflammatory and vascular protective implications [54]. A concurrent metabolic adjustment includes the remodeling of glycerophospholipids. In a cohort of patients with newly diagnosed T2DM, 12 weeks of treatment with dulaglutide or liraglutide induced significant changes in 46 and 45 metabolites, respectively, predominantly involving phosphatidylcholines, phosphatidylethanolamines, phosphatidylserines, phosphatidylinositols, and phosphatidylglycerols [82].

The observed decrease in ceramides, sphingomyelins, and lysophosphatidylcholines induced by liraglutide [54] is consistent with the mitochondrial and inflammatory mechanisms implicated in muscle metabolic health. Ceramides have been demonstrated to impair insulin signaling, and at the mitochondrial level, deplete coenzyme Q and destabilize the respiratory chain supercomplexes, leading to mitochondrial dysfunction and oxidative stress in skeletal muscle [83,84].

The GLP-1RA-induced reduction in ceramide species provides a direct metabolomic correlation—rather than mere circumstantial support—for the AMPK/SIRT1/PGC-1α-mediated improvement in mitochondrial ultrastructure and function. This reinforces, rather than merely supports, the mechanistic hypothesis.

Amino acid metabolism, particularly that of branched-chain amino acids (BCAAs), plays a central role in the regulation of insulin sensitivity and energy homeostasis. Two controlled human studies have characterized changes in the circulating amino acid profile in response to weight loss and treatment with GLP-1RAs.

Engelbrechtsen et al. (2016) [53] conducted a study on 58 obese subjects who underwent a 12% weight reduction over 8 weeks, followed by 52 weeks of weight maintenance with or without liraglutide 1.2 mg/day. The weight loss intervention led to substantial reductions (9–20%) in the plasma concentrations of alanine, phenylalanine, histidine, tyrosine, and the BCAAs leucine, isoleucine, and valine (*p* < 0.05). During the maintenance phase, valine levels were lower in the control group than in the liraglutide group (*p* = 0.005), indicating a differential modulation of BCAA metabolism by the GLP-1RAs. Concurrently, plasma citrate levels exhibited an increase, showing an inverse correlation with HOMA-IR (r = −0.318; *p* = 0.025).

In the study by Jendle et al. (2021) [54], 62 patients with T2DM were randomly assigned to receive liraglutide (up to 1.8 mg/day) or glimepiride (up to 4 mg/day) for a period of 18 weeks. Liraglutide exhibited a more pronounced effect on the metabolome, with reductions in metabolites associated with insulin resistance—including 2-hydroxybutyrate—which, conversely, increased with glimepiride. The amino acid profile associated with liraglutide was consistent with improved insulin sensitivity.

Taken together, these data indicate that weight reduction elicits a systematic decrease in BCAAs and gluconeogenic amino acids. Conversely, treatment with GLP-1RAs selectively regulates this adaptation, thereby contributing to a metabolic milieu characterized by diminished lipotoxicity and enhanced mitochondrial function.

Lower circulating BCAA concentrations during weight loss may reflect changes in dietary intake, insulin sensitivity, hepatic metabolism, or substrate oxidation. Circulating concentrations alone do not establish the direction of skeletal-muscle protein turnover. In the studies summarized here, lower BCAA concentrations occurred alongside a reduction in lean mass observed in some clinical trials. The reduction in lean mass is generally modest compared to the loss of fat mass. It does not, in itself, imply a pathological catabolic state; rather, it is indicative of a remodeling of body composition that is typical of weight loss [51]. Studies using tracers or nitrogen balance measurements are needed to determine the specific contribution of muscle protein metabolism to the described metabolomic changes. Each class of metabolites corresponds to a specific node in the molecular signaling network, and each has verifiable implications for muscle function outcomes. Making these connections explicitly clarifies where the metabolomic evidence supports the proposed mechanistic hypotheses, where it remains neutral, and where it actively challenges them.

The GLP-1RA-induced reduction in circulating BCAAs is mechanistically consistent with AMPK-mediated suppression of mTORC1 signaling: BCAAs, and leucine in particular, are themselves potent activators of mTORC1-dependent protein synthesis and of PGC-1α-driven mitochondrial biogenesis [85,86]. Consequently, a reduction in the circulating concentration of BCAA could be interpreted in two divergent ways: The first possibility is the presence of a favorable systemic signature that reflects improved insulin sensitivity and reduced amino-acid oxidation, consistent with the anti-catabolic, insulin-sensitizing properties of GLP-1RAs. The second possibility involves the role of a signal that functions in opposition to the anabolic hypothesis, as lower substrate availability for BCAA-driven mTORC1/PGC-1α activation could potentially impede anabolic and mitochondrial-biogenic signaling in muscle.

The analysis of population-level data has contributed to the resolution of some ambiguity. For instance, circulating BCAAs have been observed to be positively associated with appendicular lean mass and hand-grip strength. In addition, low circulating valine has been identified as an independent correlate of increased sarcopenia risk in a large community cohort [87]. Similarly, BCAA supplementation has been shown to acutely activate mTORC1 signaling and myofibrillar protein synthesis in human skeletal muscle [85]. This finding lends support to the hypothesis that the decline in BCAA associated with GLP-1RA therapy is more likely indicative of a decrease in dietary and energy intake, as well as an alteration in hepatic amino acid processing, rather than a signal of preserved or enhanced anabolic signaling in muscle. This distinction cannot be resolved by molecular data alone, and it underscores the need for caution in interpreting the decrease in BCAA as evidence of “metabolic quality” without the incorporation of functional measurements.

In addition, the increase in plasma citrate reported by Engelbrechtsen et al. [53], which exhibited an inverse correlation with HOMA-IR, is consistent with enhanced oxidative flux through the AMPK–PGC-1α axis. However, this relationship should be interpreted with caution regarding its clinical translation. In populations with compromised muscle mass, higher (not lower) circulating citrate has recently been shown to correlate independently with lower muscle mass and reduced hand-grip strength. This finding is attributed to disturbed mitochondrial flexibility rather than to enhanced oxidative capacity [77].

This apparent discrepancy underscores that circulating citrate is a systemic, not muscle-specific, readout, and that its relationship to muscle mitochondrial health may be context- and disease-state-dependent. This caveat should temper any direct extrapolation from the citrate changes reported in GLP-1RA trials to a presumed improvement in muscle bioenergetic capacity. A comprehensive metabolomic profiling study in aging cohorts has validated the efficacy of panels of circulating metabolites (including amino acid and lipid species analogous to those altered by GLP-1RAs) in predicting directly measured skeletal-muscle mitochondrial oxidative capacity and downstream motor function. This finding provides a framework for future GLP-1RA studies to formally link metabolomic remodeling to muscle bioenergetics and physical performance, thereby transcending the current paradigm of treating these two aspects as parallel but disconnected lines of evidence [88].

However, none of the metabolomic studies reviewed above included concurrent measurement of muscle strength, physical performance, or muscle mitochondrial function. This remains the principal limitation preventing a direct, quantitative link between the metabolomic and clinical evidence presented in this review. Nonetheless, indirect convergence exists: the metabolomic pattern associated with GLP-1RA treatment (lower BCAAs and gluconeogenic amino acids, reduced ceramides, increased citrate) mirrors the pattern that in other clinical populations correlates with preserved insulin sensitivity but not necessarily with preserved muscle strength [77,87]. This finding serves to reinforce the interpretation that enhancements in metabolic quality, as documented by metabolomics, should not be assumed to equate to the preservation of muscle function. Furthermore, this finding strengthens the call for future GLP-1RA trials to pair metabolomic profiling with objective functional endpoints, such as hand-grip strength, chair-stand testing, gait speed, and DXA-derived appendicular lean mass, within the same cohort and time frame. This approach would allow for the formal integration of mechanistic, metabolomic, and functional data rather than their juxtaposition.

## 5. Clinical Evidence

Throughout this review, lean mass denotes the method-specific non-fat compartment measured by DXA or BIA and is not used as a synonym for skeletal-muscle mass. Clinical evidence is the most relevant source for understanding the impact of GLP-1RAs and GLP-1/GIP dual agonists on lean mass, muscle function, and sarcopenia-related risk. These drugs were developed for T2DM and obesity, but are now used for other conditions. This makes it necessary to carefully evaluate the quality of the weight loss they induce.

The data show a complex picture. GLP-1RAs improve insulin sensitivity, systemic inflammation, and the cardiometabolic profile. However, a significant portion of weight loss stems from a reduction in lean mass, which may impact strength and physical performance. Because lean mass is not equivalent to skeletal muscle, these implications require separate evaluation. This section summarizes the main clinical evidence from randomized trials, observational studies, and meta-analyses.

Table 2 provides a synopsis of the findings from the clinical evidence that has been documented in the extant literature. Collectively, these studies suggest that a progressively broader degree of receptor activation (GLP-1 alone → dual GIP/GLP-1 combination → triple GIP/GLP-1/glucagon combination) corresponds to a progressively increase in weight loss. Moreover, data from the STEP 4 study underscore that these effects depend on continued pharmacological treatment and do not persist after therapy cessation.

Each trial included in Table 2 is subject to limitations that render them amenable to only a muscle-specific interpretation. The STEP 1 body-composition substudy [31] was exploratory in nature and was not powered to detect differences in lean-mass endpoints. The DXA substudy of SURMOUNT-1 [38] included a small subsample (n = 160) relative to the parent trial and lacked a matched, weight-loss-controlled comparator group. The REtatrutide Phase 2 trial [33] did not include DXA or MRI-based body-composition assessment. A review of the existing literature reveals that none of the trials listed used muscle strength or physical performance as a primary endpoint. Furthermore, there is an absence of studies that directly compared semaglutide and tirzepatide using muscle-specific outcomes in a head-to-head design.

### 5.1. Effects on Lean Mass, Fat Mass, and Muscle-to-Fat Ratio

Randomized clinical trials have consistently shown that weight loss induced by GLP-1RAs results primarily from a reduction in fat mass, but also includes a variable proportion of lean mass. In the initial trial (STEP 1), the administration of semaglutide 2.4 mg led to a mean weight reduction of 14.9% over a period of 68 weeks. In a subanalysis of body composition, approximately one-third of the weight lost (≈35%) was found to be attributable to a reduction in DXA-derived lean mass [31]. Concurrent findings were reported by Rubino et al. (2021) [37], who observed a reduction in lean mass accounting for 20% to 30% of total weight loss, with an absolute decrease of 3–5% of body weight. DXA- and BIA-derived lean-mass changes may partly reflect shifts in hydration and glycogen and should not be interpreted as direct estimates of skeletal-muscle loss [31].

These values are consistent with those observed in other contexts of rapid weight loss, such as low-calorie diets or bariatric surgery, suggesting that lean-mass loss is largely a physiological phenomenon linked to an energy deficit.

However, the rapidity and magnitude of weight reduction induced by GLP-1RAs can amplify this phenomenon, particularly among more vulnerable individuals. A comparative analysis of the SURPASS 2 study data revealed that tirzepatide demonstrates a more pronounced reduction in body weight when compared to semaglutide. However, SURPASS-2 did not include a direct head-to-head body-composition assessment [15]. In the separate SURMOUNT-1 DXA substudy (n = 160), fat mass decreased more than lean mass during tirzepatide treatment [29]. Overall, GLP-1RAs and tirzepatide result in a reduction in lean mass proportional to weight loss, although the lean-to-fat mass ratio improved because fat mass decreased more than lean mass. This finding does not by itself demonstrate the preservation of skeletal muscle or function; confirmation requires appendicular lean-mass assessment or direct muscle imaging interpreted alongside strength and physical-performance outcomes.

### 5.2. Impact on Muscle Strength and Physical Performance

Despite a documented reduction in lean mass, a significant decline in muscle strength or physical performance was not reported in the majority of clinical trials. In STEP 1, the reported physical-function outcomes were patient-reported (SF-36v2 and IWQOL-Lite-CT) rather than direct measures of muscle strength or physical performance [22]. Future trials should prioritize objective endpoints such as handgrip strength, chair-stand testing, gait speed, and the Short Physical Performance Battery.

For tirzepatide, the existing data are more limited, but the available studies do not indicate a significant functional decline. However, the greater extent of weight loss could pose a risk in older or frail individuals, in whom even a modest reduction in lean mass could have significant clinical consequences. The observed loss of lean mass by GLP-1RAs has given rise to sarcopenia-related concern, particularly among vulnerable populations such as the elderly, individuals with sarcopenic obesity, or patients with pre-existing frailty. In these individuals, even a modest reduction in skeletal muscle mass, when accompanied by lower strength or performance, can compromise functional reserve, increase the risk of falls, and accelerate progression toward frailty [34].

### 5.3. Sarcopenia-Related Risk and Frailty: Older Adults, Sarcopenic Obesity, and Type 2 Diabetes Mellitus (T2DM)

The impact of GLP-1RAs on lean mass may exhibit variability depending on the characteristics of the treated population. It has been demonstrated that the protein synthesis capacity of older adults is diminished, rendering them more susceptible to muscle loss. Recent data suggest that GLP-1RA-associated lean-mass loss, although heterogeneous, may be a particularly significant concern in older or frail individuals, necessitating the careful monitoring of body composition and muscle function and a more cautious therapeutic approach [89]. In clinical practice, a baseline assessment of patients at elevated sarcopenia risk should encompass the measurement of handgrip strength (low-strength threshold: <27 kg in men, <16 kg in women) or the five-times chair-stand test (>15 s considered indicative of impaired lower-limb performance), in accordance with the EWGSOP2 case-finding criteria [90]. During treatment, the appendicular skeletal muscle mass index (ASMI) should be reassessed approximately every 3–6 months via DXA or BIA in high-risk patients, using EWGSOP2 reference cut-points for confirmed low muscle mass (ASMI < 7.0 kg/m^2^ in men, <5.5 kg/m^2^ in women by DXA) as an actionable trigger for intensified nutritional and exercise intervention [90]. Concurrently, the recommended daily protein intake is a minimum of 1.2 g/kg (up to 1.5–1.6 g/kg where appropriate) of ideal or adjusted body weight. In addition, resistance training should be prescribed at a minimum of 2–3 sessions per week.

Sarcopenic obesity should be defined as excess adiposity accompanied by both reduced muscle function and altered body composition, rather than by low lean mass alone. The ESPEN/EASO diagnostic pathway uses screening followed by the assessment of muscle function with handgrip strength or a chair-stand test and confirmation of altered body composition using adiposity and muscle-mass indices appropriate for people with obesity. Because sarcopenic obesity may coexist with osteoporosis and other skeletal alterations, bone health should also be considered when evaluating older or otherwise vulnerable patients [91,92]. In individuals with sarcopenic obesity, the coexistence of excess fat mass and muscle mass deficiency makes this population particularly vulnerable to the effects of GLP-1RAs on body composition. In such cases, the patients’ muscle reserve is already diminished from the outset. Consequently, even a loss of lean mass that is proportionally similar to that observed in the general population can result in a clinically significant deterioration in physical function and an increased risk of frailty [93]. In this subgroup, the combination of drug therapy and resistance exercise is particularly important for preserving muscle function during weight loss [35]. Additionally, food-medicine homology compounds such as resveratrol may offer complementary musculoskeletal benefits, as reviewed in the context of sarcopenia, osteoporosis, and osteoarthritis [94].

In patients diagnosed with T2DM, the clinical picture is further complicated by the possible presence of diabetic neuropathy, diabetic sarcopenia, and reduced intrinsic muscle quality, regardless of body weight [95,96,97].

A recent review by Liu et al. (2024) [98] provides a comprehensive comparison and synthesis of sarcopenia, disuse muscle atrophy, and sarcopenia associated with T2DM. The review focuses on pathological changes, epidemiology, clinical presentation, etiology, and prevention and treatment strategies. T2DM-related sarcopenia differs from both age-related sarcopenia and disuse atrophy with respect to histopathological patterns (reduction in type I fibers) and the mechanisms involved (insulin resistance, inflammatory state, oxidative stress)—mechanisms that remain poorly understood. The findings reveal that these three conditions exhibit both commonalities and distinctions in terms of etiology, age-related characteristics, pathological changes, clinical manifestations, and pathophysiological mechanisms [98].

In this particular population, treatment-induced loss of lean mass could exacerbate a preexisting deficit in muscle quality associated with the metabolic disease itself [99], underscoring the necessity for comprehensive body composition monitoring (e.g., via DXA or BIA) during treatment, particularly in patients with long-standing disease or with micro- or macrovascular complications [100].

Overall, these data suggest that the effect of GLP-1RAs on lean mass should not be considered uniform but must be contextualized based on the patient’s baseline clinical phenotype, with particular caution in subgroups at combined risk (advanced age + sarcopenic obesity + T2DM). This risk-based approach should also consider the potentially different magnitude of weight loss induced by individual agents. Theoretically, tirzepatide may pose a greater risk of clinically meaningful lean-mass loss in older adults or individuals with sarcopenic obesity because of its superior weight-loss efficacy compared with that observed with lower-dose semaglutide regimens. This potential risk must be weighed against the substantial benefits of tirzepatide, which have been demonstrated to reduce glycemia and cardiometabolic risk. To date, there has been no adequately powered, weight-loss-matched head-to-head trial that has directly compared semaglutide and tirzepatide in older adults or in individuals with sarcopenic obesity. This finding underscores the significance of this area as a future research priority.

### 5.4. Explaining the Paradox: Muscle Quantity, Muscle Quality, and the Energy/Protein Deficit Hypothesis

The coexistence of favorable molecular signals and clinically documented lean-mass loss becomes less paradoxical once four dimensions are considered together.

Firstly, lean mass, as measured by DXA or BIA, is a composite compartment that includes water, glycogen and its bound water, and connective tissue, in addition to contractile protein. This compartment is distinct from skeletal muscle mass proper, as measured by CT or MRI. Controlled manipulation studies have demonstrated that glycogen depletion and fluid shifts, in isolation, can result in apparent DXA-derived lean-mass changes of 1–3%, despite the absence of any true change in myofibrillar protein [101,102]. It has been demonstrated that GLP-1RA-induced weight loss frequently results in a reduction in carbohydrate intake and glycogen stores. Consequently, a significant portion of the lean-mass loss measured by DXA/BIA, as reported in this review, may be attributable to this artifact rather than to authentic muscle-protein loss.

Secondly, the muscle fibers that persist following treatment-induced weight loss may exhibit higher functional quality. Myosteatosis, defined as ectopic fat infiltration within muscle tissue, exhibits an inverse correlation with strength and mobility, independent of muscle volume [103]. The mitochondrial-biogenic and anti-inflammatory mechanisms previously described are likely the pathways that facilitate the expected reductions in intramuscular fat infiltration and the improvements in mitochondrial density within residual muscle mass. These mechanisms offer a plausible, although not yet directly tested, explanation for the preservation of strength despite measurable lean-mass loss.

Thirdly, the magnitude of true myofibrillar protein loss appears to be modifiable by protein intake. Cross-sectional data demonstrate that the majority of GLP-1RA users do not meet the recommended protein targets [104,105]. A small case series involving patients who combined high protein intake with structured resistance training reported lean-tissue changes ranging from −6.9% to +5.8% of the baseline, which is in stark contrast to the 20–40% lean-mass-to-weight-loss ratio typically reported without such intervention [106]. This finding lends support to the hypothesis that a significant portion of the observed lean-mass loss is attributable to insufficient protein intake during periods of appetite suppression, as opposed to being a direct consequence of catabolic drug effects.

Fourthly, the quantity-versus-quality distinction and the protein-threshold hypothesis must be tested directly within GLP-1RA and dual/triple-agonist trial populations using water-independent reference methods (CT/MRI or D3-creatine dilution) alongside functional and nutritional endpoints.

### 5.5. Integrated Management Strategies in Clinical Practice

The translation of this evidence into clinical practice necessitates a systematic, three-pronged approach. A comprehensive baseline evaluation encompasses a rapid screening procedure employing the SARC-F questionnaire, a five-item, self-reported instrument validated for its effectiveness in gauging physical function, as indicated by the ability to ambulate, ascend and descend stairs, and maintain balance [107]. This evaluation should be conducted prior to the initiation of any therapeutic interventions in patients considered to be at risk. Alternatively, a handgrip dynamometry assessment can serve as a substitute for the aforementioned screening procedure, providing equivalent diagnostic insights. Nutritional support should aim to achieve an intake of protein of at least 1.2–1.5 g/kg of ideal body weight per day. Meta-analytic evidence indicates that β-hydroxy-β-methylbutyrate (HMB) supplementation can reduce lean-mass loss in elderly individuals, although its effect on strength and physical performance is less consistent across trials [108]. Therefore, it should be regarded as a plausible adjunct rather than a standard of care in this specific population. Exercise prescription should emphasize progressive resistance training, the most effective non-pharmacological intervention for preserving muscle mass during energy-restricted weight loss, as a standard component of care rather than an optional adjunct [106].

## 6. Limitations of the Review

This review has some methodological limitations that should be taken into account when interpreting the results.

First, the synthesis was conducted primarily as a narrative review rather than through quantitative meta-analysis, given the heterogeneity in study design, patients populations, treatment duration, assessment of body composition (DXA, BIA, CT), and the outcome measures, and this limits the ability to estimate an aggregate effect size for lean mass loss associated with the various incretin agonists.

Second, most of the available clinical data on body composition derive from subanalyses of trials primarily designed to assess weight loss or glycemic control, not the quality of weight loss, which may increase the risk of outcome reporting bias and limits the statistical robustness of comparisons between drugs. Most studies also lacked weight-loss-matched comparator groups, limiting the separation of drug-specific effects from changes mediated by energy deficit and weight reduction.

Third, the direct comparison between semaglutide and tirzepatide is limited by the absence of adequately powered head-to-head RCTs with muscle-related endpoints as the primary outcome, making comparative conclusions preliminary.

Finally, much of the mechanistic evidence discussed in this review derives from preclinical or cellular models. Consequently, its translational relevance to humans remains uncertain, particularly with respect to the existence and functional significance of GLP-1 signaling in human skeletal muscle, an area that remains under investigation and requires further clinical and mechanistic studies.

Several priority questions remain unresolved by the current evidence base. Firstly, there is a significant absence of long-term follow-up data that address the fracture risk or the interaction between bone and muscle in older adults undergoing sustained incretin-based therapy. Secondly, there is a paucity of adequately powered, weight-loss-matched, head-to-head trials comparing semaglutide, tirzepatide, and retatrutide using muscle-specific imaging and functional endpoints—rather than body weight alone—particularly in older or sarcopenic-obese populations. Thirdly, the heterogeneity in sarcopenia definitions and body-composition assessment methods (DXA vs. BIA vs. CT/MRI) across studies limits the comparability of results; therefore, the adoption of standardized, muscle-quality-inclusive protocols is a priority. Fourthly, prospective studies directly linking treatment-induced changes in intramuscular fat infiltration and mitochondrial density to functional outcomes are needed to test the muscle-quality hypothesis previously discussed. Finally, the primary research objectives for the future include conducting dose-finding trials that are specifically designed to test the effects of protein or HMB supplementation during GLP-1RA therapy. Additionally, the research agenda entails the implementation of dedicated body-composition substudies for Retatrutide at its full efficacy range.

## 7. Conclusions

This review highlights a complex and multidimensional picture of the effects of GLP-1RAs, dual GLP-1/GIP agonists, and the more recent triple GLP-1/GIP/glucagon agonists on skeletal muscle. An integrated analysis of preclinical, metabolomic, and clinical evidence reveals an apparent paradox: potentially protective molecular signals coexist with a clinical loss of lean mass—sometimes significant—observed in trials conducted with semaglutide and tirzepatide.

At the molecular level, the activation of GLP-1R and GIPR in skeletal muscle is associated with predominantly favorable signals: enhanced glucose uptake via GLUT4, activation of mitochondrial pathways (AMPK/SIRT1/PGC-1α), and reduced inflammatory and oxidative stress. These mechanisms, when considered in isolation, would suggest a neutral or protective effect on muscle mass.

This apparent discrepancy requires a critical and integrated evaluation that considers the biological mechanisms together with nutritional status, energy balance, and clinical characteristics of the treated patients.

A substantial portion of the loss of lean mass may reflect the magnitude and rate of weight loss, reduced energy or protein intake, and gastrointestinal intolerance; however, current trials do not allow these mechanisms to be clearly separated from drug-specific effects [59].

In summary, incretin agonists represent a class of drugs that is extraordinarily effective in the treatment of obesity and T2DM, but their impact on muscle health requires a balanced interpretation. The protective molecular effects alone are not sufficient to prevent lean mass loss under conditions of energy deficit. Lean-mass loss alone, however, should not be interpreted as skeletal-muscle loss or sarcopenia. The quality of weight loss, rather than the weight loss alone, must become a central parameter in clinical evaluation, especially in populations at risk for sarcopenia. An integrated approach—pharmacological, nutritional, and physical—is essential to maximize the benefits and minimize the muscle-related risks associated with these therapies.

Practical monitoring during incretin-based therapy. At the baseline, clinicians should assess nutritional status, dietary intake, gastrointestinal history, muscle strength and function, and, when clinically indicated, body composition. During dose escalation and follow-up, monitoring should include dietary adequacy, gastrointestinal tolerance, the rate of weight loss, and changes in strength, performance, and body composition. Protein intake and resistance exercise should be individualized according to age, frailty, habitual activity, comorbidities, and renal function [109].

## Figures and Tables

**Table 1:** Structural characterization and pharmacokinetic evolution of endogenous incretins and their synthetic analogues: comparative analysis of primary amino acid sequences, engineering modifications, and mono-, dual-, and triple-receptor activation profiles.

Feature	Native GIP [2,3]	Native GLP-1 [1,2,3]	Liraglutide [18,19]	Semaglutide [20,21]	Dulaglutide [22,23]	Tirzepatide [24,25]	Retatrutide [26,27]
Number of amino acid residues	42	30	31	31	31-aa GLP-1 analogue per chain (+ 16-aa linker + modified IgG4 Fc)	39	39
Primary structure	Tyr-Ala-Glu-Gly-Thr-Phe-Ile-Ser-Asp-Tyr-Ser-Ile-Ala-Met-Asp-Lys-Ile-His-Gln-Gln-Asp-Phe-Val-Asn-Trp-Leu-Leu-Ala-Gln-Lys-Gly-Lys-Lys-Asn-Asp-Trp-Lys-His-Asn-Ile-Thr-Gln	His-Ala-Glu-Gly-Thr-Phe-Thr-Ser-Asp-Val-Ser-Ser-Tyr-Leu-Glu-Gly-Gln-Ala-Ala-Lys-Glu-Phe-Ile-Ala-Trp-Leu-Val-Lys-Gly-Arg	His-Ala-Glu-Gly-Thr-Phe-Thr-Ser-Asp-Val-Ser-Ser-Tyr-Leu-Glu-Gly-Gln-Ala-Ala-Lys *-Glu-Phe-Ile-Ala-Trp-Leu-Val-Arg-Gly-Arg-Gly	His-Aib-Glu-Gly-Thr-Phe-Thr-Ser-Asp-Val-Ser-Ser-Tyr-Leu-Glu-Gly-Gln-Ala-Ala-Lys *-Glu-Phe-Ile-Ala-Trp-Leu-Val-Arg-Gly-Arg-Gly	His-Gly-Glu-Gly-Thr-Phe-Thr-Ser-Asp-Val-Ser-Ser-Tyr-Leu-Glu-Glu-Gln-Ala-Ala-Lys-Glu-Phe-Ile-Ala-Trp-Leu-Val-Lys-Gly-Gly-Gly	Tyr-Aib-Glu-Gly-Thr-Phe-Thr-Ser-Asp-Tyr-Ser-Ile-Aib-Leu-Asp-Lys-Ile-Ala-Gln-Lys *-Ala-Phe-Val-Gln-Trp-Leu-Ile-Ala-Gly-Gly-Pro-Ser-Ser-Gly-Ala-Pro-Pro-Pro-Ser	Tyr-Aib-Gln-Gly-Thr-Phe-Thr-Ser-Asp-Tyr-Ser-Ile-aMeLeu-Leu-Asp-Lys-Lys *-Ala-Gln-Aib-Ala-Phe-Ile-Glu-Tyr-Leu-Leu-Glu-Gly-Gly-Pro-Ser-Ser-Gly-Ala-Pro-Pro-Pro-Ser
N-Terminus	Tyr	His	His	His	His	Tyr	Tyr
Position 2 modification	None (Ala)	None (Ala)	None (Ala)	Aib	Gly	Aib	Aib
Other modifications	-	-	Pos. 34: Arg	Pos. 34: Arg	Pos. 16: Glu; Pos. 30: Gly	Pos. 13: Aib; Engineered C-terminal extension	Pos. 13: α-Me-Leu; Pos. 20: Aib
Fatty acid chain	-	-	C16 palmitoyl via gamma-Glu spacer on Lys26	C18 fatty diacid via hydrophilic spacer on Lys26	-	C20 fatty diacid via linker on Lys20	C20 fatty diacid via linker on Lys17
Protein ballast	-	-	-	-	Modified IgG4 Fc fusion	-	-
C-Terminus	Free (-COOH)	Amidated (-NH_2_)	Free (-COOH)	Free (-COOH)	Covalently linked via peptide linker to modified IgG4 Fc	Amidated (-NH_2_)	Amidated (-NH_2_)
Receptor target	GIPR only	GLP-1R only	GLP-1R only	GLP-1R only	GLP-1R only	Dual (GIPR/GLP-1R)	Triple (GIPR/GLP-1R/GCGR)
Biological half-life	5–7 min	~1.5–2 min	~13 h	~7 days	~5 days	~5 days	~6 days
Dosing frequency	Not applicable (endogenous secretion)	Not applicable (endogenous secretion)	Once daily	Once weekly	Once weekly	Once weekly	Once weekly

Explanatory notes to the table: 1. Lipid anchoring (*): The asterisk shown in the primary-structure row (e.g., Lys *) identifies the exact lysine residue used for acylation. The fatty acid chain attached at this site acts as a reversible “hook” for plasma albumin, protecting the drug from renal filtration. 2. DPP-4 resistance (position 2): Native hormones contain alanine at position 2, which makes them immediate substrates for the degradative enzyme DPP-4. Substitution with synthetic or alternative amino acids, such as Aib (α-aminoisobutyric acid) or glycine, chemically prevents enzymatic cleavage. 3. Evolution of the C-terminus: The proline- and serine-rich C-terminal extensions of tirzepatide and retatrutide are components of their engineered peptide pharmacophores. Both molecules are C-terminally amidated (-NH_2_). 4. N-terminal selectivity: The initial amino acid contributes to baseline receptor affinity. Histidine (His) favors binding to the GLP-1 receptor, whereas tyrosine (Tyr) is essential for activation of the GIP receptor. 5. Co-agonist engineering: Targeted substitutions along the peptide chain, such as α-Me-Leu at position 13 in retatrutide, modify three-dimensional structural flexibility, enabling a single molecule to fit into and activate up to three different receptors: GIPR, GLP-1R, and GCGR. 6. Molecular strategy of dulaglutide: Unlike other analogues acylated with fatty acids, dulaglutide prolongs its half-life by covalently linking the peptide to a large protein ballast, namely the Fc fragment of an IgG4 immunoglobulin, thereby exceeding the renal clearance threshold through steric hindrance. 7. Sources: Native GIP and GLP-1 [1,2,3]; liraglutide [18,19]; semaglutide [20,21]; dulaglutide [22,23]; tirzepatide [24,25]; retatrutide [26,27]. Sequences are shown for the peptide pharmacophore; dulaglutide is a dimeric Fc-fusion protein, and the lipidated analogues include the spacers/linkers specified in the cited primary sources. Half-live (values) are approximate clinical values.

**Table 2:** Summary of Key Clinical Trial Evidence.

Drug	Study/Design	Dosage	Patients (N Population)	Key Finding	Refs.
**Tirzepatide vs. Semaglutide**	SURPASS-2—phase 3 RCT, open-label, 40 weeks	Tirzepatide 5/10/15 mg SC/week vs. Semaglutide 1 mg SC/week	N = 1879, adults with T2D on metformin (baseline HbA1c 8.28%; weight 93.7 kg)	Tirzepatide superior to semaglutide for HbA1c and weight reduction at all doses	Frías et al. 2021 [15]
**Semaglutide**	STEP 1—phase 3 RCT, double-blind, 68 weeks	2.4 mg SC/week vs. placebo	N = 1961 (1306 semaglutide, 655 placebo), overweight/obesity without diabetes	Mean weight loss ~14.9% vs. 2.4% with placebo	Wilding et al. 2021 [31,32]
**Semaglutide** (body composition)	STEP 1—exploratory analysis (DXA substudy)	2.4 mg SC/week	Subsample of STEP 1	Fat mass reduction greater than lean mass reduction	Wilding et al. 2021 [31,32]
**Semaglutide**	STEP 4—phase 3 RCT, weight maintenance, 68 weeks (20-week run-in + 48-week randomized period)	2.4 mg SC/week, continued vs. withdrawn (switch to placebo)	N = 902 in run-in → 803 randomized (535 semaglutide, 268 placebo)	Continued treatment maintains weight loss (−7.9% vs. +6.9% upon withdrawal)	Rubino et al. 2021 [37]
**Tirzepatide**	SURMOUNT-1—phase 3 RCT, double-blind, 72 weeks	5/10/15 mg SC/week vs. placebo	N = 2539, obesity/overweight without diabetes	Weight loss 16.0–22.5% depending on dose	Jastreboff et al. 2022 [33]
**Tirzepatide** (body composition)	SURMOUNT-1—DXA substudy	5/10/15 mg SC/week	Subsample of SURMOUNT-1	Fat mass reduction ~3 × greater than lean mass reduction (33.9% vs. 10.9%)	Look et al. 2025 [38]
**Retatrutide** (triple GIP/GLP-1/glucagon agonist)	Phase 2, dose-ranging, double-blind, 48 weeks	1/4/8/12 mg SC/week vs. placebo	N = 338, obesity/overweight without diabetes	Weight loss up to 24.2% with the 12 mg dose	Jastreboff et al. 2023 [26]

## Data Availability

No new data were created or analyzed in this study. Data sharing is not applicable to this article.
